# A Cross-Sectional Survey on Professionals to Assess Health Needs of Newly Arrived Migrants in Spain

**DOI:** 10.3389/fpubh.2021.667251

**Published:** 2021-08-02

**Authors:** Núria Serre-Delcor, Inés Oliveira, Ruben Moreno, Begoña Treviño, Eva Hajdók, Esperanza Esteban, Adrià Murias-Closas, Abdallah Denial, Stella Evangelidou

**Affiliations:** ^1^Tropical Medicine and International Health Unit Drassanes-Vall d'Hebron, PROSICS (International Health Program ot the Catalan Health Institute), Department of Infectious Diseases, Hospital Vall d'Hebron, Institut Català de la Salut, Barcelona, Spain; ^2^Vall d'Hebron Research Institute, Infectious Diseases Group, Barcelona, Spain

**Keywords:** migrant, healthcare, social care, barriers, needs, survey, professionals

## Abstract

Heightened conflicts and lack of safety due to reasons related to economic, social, ethnic, religious, sexual orientation, political, or nationality matters have increased migratory movements during the last, few decades. Unfortunately, when migrants arrive in new territories, they can face many barriers. For example, in Spain, some migrants have difficulties in accessing health services. The main objective of this study was to describe, from the perspective of social and healthcare professionals, health needs and barriers faced among migrants who recently arrived in Spain when accessing the health system. To accomplish this aim, we carried out a cross-sectional descriptive study using a newly created self-administered questionnaire. Statistical analysis was done using the SPSS 23.00^®^ program. Survey collection was from April 2018 to October 2018, and the cohort comprised a total of 228 professionals. Most participants were females (76%), with an average age of 35 years [interquartile range (IQR) 29.8–43.0]. The most represented profession in the cohort was physician (48%), followed by social care professionals (32%), nursing (11%), and other (8%). Of these individuals, 61% stated having either little or limited knowledge of international migrant health rights, and 94% believed migrants must overcome barriers to receive health services. The four most reported barriers were as follows: language, cultural differences, administrative issues, and fear of being undocumented. Additionally, by order of importance, professionals viewed mental health disorders and infectious diseases as the most common contributors to disease burden in this group. The four most popular strategies implemented by professionals to improve healthcare access further for migrants included intercultural competency training for professionals; access to community health agents; access to translators; and development of health system navigation skills among those newly arrived. Study results suggest that governments should make greater efforts to provide social and healthcare professionals with more effective tools that overcome communication barriers and cultural competence training modules.

## Introduction

Heightened conflicts and lack of safety due to reasons related—yet not limited to—to economic, social, ethnic, religious, sexual orientation, political, or nationality matters have increased migratory movements during the last, few decades (1). According to the International Organization for Migration (IOM), there were 272 million international migrants globally (comprising 3.5% of the total world population) in 2019. Additionally, in 2017, there were 68.5 million displaced individuals in the world, of whom 25.5 million were refugees. In absolute number, the most common destinations for migrants in 2019 included Asia, Europe and North America (1).

Indeed, according to Eurostat, there were 21.8 million international migrants across the 27 European Union member states (EU-27), constituting 4.9% of the EU-27 total population by January 2019. Spain ranked as the fifth country in hosting the highest percentage (13.8%) of such migrants (2) and the major community by area of origin are Latin Americans (LA). The flow of LA to Spain started in the 1970 s mainly for historical and political agreements. However, the largest number of them came in the mid-1990 s, with the arrival of women seeking domestic work. In the same decade, there was a significant increase in migrants coming from Africa, mostly Moroccans, due to geographic proximity and job opportunities in the agricultural and construction areas. They became the second largest continent of origin after LA (3). Nowadays, the three largest migrants groups in Spain by country of origin are Morocco, Romania and Ecuador (4); however, according to recent data provided by the European Center for Disease Prevention and Control, Venezuela is the most common country of origin among asylum seekers in Spain (5). Furthermore, the flow of irregular migrants to Europe has dramatically increased since 2015 (6). A study from the University of Carlos III and Causa Foundation estimated that between 390,000 and 470,000 irregular migrants (80% LA and 10% Africans) were present in Spain at the end of 2019, representing a 6 to 20-fold increase since 2014. However, this absolute number accounts for only 0.8% of the total population (7). Despite the fact that migrants have been proven to contribute to host countries' economy growth (8) and different studies (9–11) have demonstrated the “healthy migrant effect” shortly after arrival (migrants are generally healthier than native-born populations partly due to a self-selection process prior to migration), some areas of society continue expressing a concern that the migrant community could incur additional costs for host countries (12, 13). However, a 2016 systematic review on 36 publications (of which 28 were from Europe) exploring differences in the use of health services between migrants and native populations concluded that migrants generally take advantage of health services less frequently (14). Interestingly, although irregular migrants are often equated to health tourism, a survey conducted by Medicus Mundi in Spain showed that only 4% of this particular group came due to medical reasons. This practice was indeed far more common for foreign populations coming from within the European Union (15).

Nonetheless, when migrants newly arrive to a territory within the EU-27, they are met with various barriers when accessing health services. Countries throughout the EU-27 have significantly different entitlement regulations for irregular migrants with respect to health systems (16). For example, Germany, Denmark, or Belgium only provide access to emergency care and certain services for specific conditions to irregular migrants (17). In other countries like Sweden, Slovenia, the UK, Croatia, and Germany, healthcare providers must report irregular migrants, which dissuades some individuals from seeking the health assistance needed (16). In Catalonia (Spain) all residents are guaranteed healthcare (18); however, some social entities work for the actual application of this law (19). Another aspect worth considering is the existing sociocultural difficulties, too. The primary obstacle for this population is the insufficient knowledge of the host country's language to comprehend how to benefit from the health system and how to explain symptoms or health needs (20). Lastly, other potential changes for the migrant population include a low level of education and health literacy; the high complexity of bureaucracy and legal channels; and, the lack of socioeconomic means to afford co-payments or even attend an appointment (21).

At the same time, migrants' health status will be largely determined by their epidemiological origin, the migratory process, genetics, and other social determinants of health. According to the IOM, varying social determinants of health, such as socioeconomic or work conditions, elevate migrants' exposure to health risks. Oftentimes, this host of situations proves worse for women, minors, and less qualified migrants, especially those in an irregular administrative standing. In 2009, IOM recognized the importance of addressing migration as a social determinant of health for migrants (22).

That stated, between April 2017 and June 2020, the Health Program of the European Union Consumers, Health, Agriculture and Food Executive Agency (CHAFEA) co-funded the MyHealth project (Models to Engage Vulnerable Migrants and Refugees in their Health, through Community Empowerment and Learning Alliance; http://www.healthonthemove.net/). The general aim of MyHealth was to improve healthcare access for vulnerable, newly arrived migrants and refugees (VMR) in Europe by developing and implementing models based on the knowhow of a European multidisciplinary network. To that aim, one of the first tasks into the project was to provide key aspects of the needs of migrants in terms of health. Different approaches were used for that task (literature review, focus groups, individual interviews, and online questionnaires). The health needs detected were later used to define and develop health appropriate strategies/tools (workshops, trainings, leaflets, audio-visuals materials, guidelines, etc).

As part of this project, the manuscript presented herein has the main objective to describe, from the perspective of social and healthcare professionals, health needs and barriers faced among migrants who recently arrived in Spain when accessing the health system.

## Materials and Methods

We conducted a cross-sectional descriptive study based on convenience sampling using a newly created self-administered questionnaire aimed at social and healthcare professionals attending to recently arrived migrants. Recently arrived migrants were defined as those individuals who arrived to Europe from a non-EU-27 country within the last 5 years (23).

After completing a brief literature review, we did not find relevant examples of validated surveys (with closed-ended questions) that assessed the health needs and barriers among recently arrived migrants to Spain from the perspective of social and healthcare professionals. Consequently, we designed our questionnaire by combining both a top-down and bottom-up approach to survey building that involved several steps. First, we asked several field experts to come up a broad list of items on the health-needs and barriers among recently arrived migrants to Spain. Second, we carried out several focus groups, with healthcare (*n* = 8), social care professionals (*n* = 8), and with migrants (*n* = 19), with the purpose of including the broader community-based perspective, resulting in a first questionnaire draft of 50-item. Third, for the purpose of reducing the overall length of the questionnaire we tasked a panel of experts to narrow down the number of items taking into account mostly redundancy, relevancy, and appropriateness. In addition, one community health agent and one professional who were not directly related to the research also participated in the questionnaire creation process by providing feed-back on the content and face-validity of the questions. Lastly, the final version of the questionnaire included 22 items, with an average completion time of 7 min. Close-ended questions included sociodemographic information (gender, age, and country of origin); job profile (type of workplace, profession, and years of experience); perceptions of quality of health in migrants; health access information; barriers; and possible solutions (see Supplementary Material).

The survey and research information were available online in Spanish using Survey Monkey©. Participation was voluntary and anonymous, and no personal or identifiable data were collected. Consent was assumed if the survey was completed. The online survey was offered through MyHealth website at the III Workshop for social care professionals on psychosocial and communicable diseases in asylum seekers and migrants in vulnerable situation in Barcelona on April 2018 (*n* = 150), at the International Conference of Migration and Health in Rome on Oct 2018 (*n* = 100), and at the Master of International Health of the Universitat Autonoma de Barcelona on June 2018 (*n* = 40). In addition, MyHealth stakeholders (*n* = 85) received a personal invitation to the survey. All participants were invited to share the online survey to their workmates. The data collection period was from April 2018 to October 2018.

The study was carried out in accordance with the Harmonized Tripartite Standards for Good Clinical Practice, following current national regulations (Law 14/2007 of Biomedical Research) and the Ethical principles derived from the Declaration of Helsinki. Data confidentiality of study participants was guaranteed in compliance with the Regulation EU 2016/679 of the European Parliament and of the Council of April 27, 2016 on Data Protection (RGPD). This research was evaluated and approved by the Clinical Research Ethics Committee (CEIC) of the Vall d'Hebron Hospital in Barcelona and adhered to best clinical practices.

Statistical analysis included measurements on distribution, central tendency (median or average if standard deviation was >20%), and dispersion [standard deviation (SD) and interquartile range (IQR)]. The statistical analysis was carried out using the SPSS 23.00^®^ program (IBM Corp. 2015, Armonk, NY).

## Results

A total of 228 professionals completed the survey. Since ~375 professionals were invited to participate (85 personally and 290 at conferences), we estimate that 60% of those invited responded to our call to participate.

### Demographics and Work Profile

In the cohort, 172 of 227 (75.8%) participants were females, with an average age of 35 years (IQR 29.8–43.0). With respect to profession, 109 of 226 (48.2%) participants were physicians; 73 (32.3%) social care professionals; 25 (11.1%) nursing professionals; and 19 (8.4%) other health professions. Regarding the place of work, 75 of 226 (33.2%) participants worked in primary healthcare centers; 65 (28.8%) hospitals; 41(18.1%) non-governmental organizations: 25 (11.1%) associations or foundations; 14 (6.2%) other types of health services; and 6 (2.7%) other types of resources (government administration or education). Work experience ranged as follows: 91 of 226 (40.3%) participants had >10 years; 80 (35.4%) <5 years; and 55 (24.3%) 5–10 years. Additionally, only 50 of 223 (22.4%) participants responded that the percentage of migrants using their services was <10%. Lastly, per participants' responses, the most common communities represented in their centers were LA, followed by sub-Saharan African, North African, Southern Indian, Eastern European, and Middle Eastern.

### Questions Concerning Healthcare Among Migrants and Refugees

In general, professionals perceived that the average health status of the native population is better than the migrant population. When ranking migrants' health status by region of origin, LA scored best, following by Eastern Europeans and Middle Easterners. Conversely, sub-Saharan African migrants were considered to have the worse level of health status.

### Perceived Use of Healthcare Services of Migrant vs. Native Populations

When comparing the use of healthcare services among migrant vs. native populations, including refugees and asylum seekers, in their respective countries, 117 of 217 (53.9%) professionals indicated the native population abused healthcare services. However, only 26 of 224 (11.6%) professionals believed migrants abused the health system.

### Perceived Knowledge of Migrants' Health Rights

Of the 225 participants who responded to the question related to migrants' health rights, 137 (60.9%) stated having either little or limited knowledge regarding such health rights. Only 66 of 228 (28.9%) participants reported acquiring knowledge of migrants' health rights through specific training provided at their workplace. An overwhelming majority of the professional cohort—212 of 226 (93.8%)—believed the migrant population faced barriers when accessing health services. The four most reported barriers were as follows: language, cultural differences, administrative issues, and fear of being undocumented (Table 1).

**Table 1:** Professionals' responses to the question, “what barriers/problems/difficulties does the migrant population face when accessing health services?

**Barrier to healthcare access**	***N* = 228**
Language	202 (88.6%)
Cultural	161 (70.6%)
Administrative issues	156 (68.4%)
Fear of being undocumented	131 (57.5%)
Work or employment	91 (39.9%)
Religious	74 (32.5%)
Stigma/prejudices	69 (30.3%)
Family obligations	55 (24.1%)
Being a man or woman	49 (21.5%)
Geographic	14 (6.1%)

### Health Burden

Relative to health-burden, 154 of 228 (67.5%) participants perceived mental health disorders as the disease most commonly present among newly arrived migrants; 126 (55.3%) infectious diseases; and 100 (43.9%) non-communicable diseases. A minority [33 of 228 (14.5%)] believed migrants were generally healthy (Table 2). Only 75 of 170 (44.1%) professionals reported receiving specific training on social or healthcare in the migrant population.

**Table 2:** Professionals' responses to the question, “In your opinion, what type of diseases is more frequent in migrants, refugees, and asylum seekers?

**Health issue**	***N* = 228**
Mental Health	154 (67.5%)
Infectious diseases	126 (55.3%)
Non-communicable diseases	100 (43.9%)
Dental health	94 (41.2%)
Migrants are healthy	33 (14.5%)

### Suggested Strategies to Improve Access to and Use of Healthcare Services

From the point of view of professionals, the following strategies were considered as the most popular when improving care for migrants, refugees, and asylum seekers: increased intercultural competency training for professionals; increased availability of community health agents and translators; and increased efforts to train migrants on using the health system (Table 3).

**Table 3:** Professionals' responses to the question, “What tools/strategies do you think could improve care for migrants, refugees, and asylum seekers in your center?”

**Strategies to improve health care**	***N* = 228**
Training professionals on intercultural health skills	145 (63.6%)
Community Health Agents	141 (61.8%)
Translators	130 (57.0%)
Training migrants on using the health system	110 (48.2%)
Education and health promotion activities for the migrant population	101 (44.3%)
Training professionals on the most prevalent diseases in migrants	81 (35.5%)
Specific referral circuits for mental health	75 (32.9%)
Specific referral circuits for international health services	71 (31.1%)
Training professionals on law regulations regarding access to the health system	62 (27.2%)
Mobile applications/internet	17 (7.5%)

## Discussion

Professionals participating in our study were mainly young adult (aged 35 years) females (76%) in the healthcare sector (68%) with more than 5 years of work experience (65%). This data slightly differ from the usual profile of health and social care professionals in Spain. According to official data, only 21% of physicians in Spain are <35 years old (24). Women represent 52% of medical doctors, 84% of nurses, and 95% of social workers in Spain (25, 26). The data of our cohort could reflect the feminization of health and social care in the last, few decades in Spain. According to official data in Spain, the percentage of men working in this field has decreased from 12.4% in 2009 to 3.7% in 2019, while the percentage of women increased from 12.4% in 2009 to 14.2% in 2019 (27). However, another possible explanation is that women and younger age have shown to be more participatory in surveys (28). The most common migrant community by area of origin at the centers were LA, which is consistent with the largest migrant population in Spain (3).

Professionals perceived the health status of the native Spanish population as better than that of newly arrived, non-EU-27 migrants. This perception could be justified, given that it matches distribution of different life expectancy figures from around the world, ranging from aged >70 years old in Europe to aged < 50 years in some African areas (29).

We oberserved that social and healthcare workers participants perceived the native Spanish population as more prone to abusing healthcare services than newly arrived migrants. This finding is compatible with other autors sharing similar data. For example, one systematic review (14) concluded that in countries with universal healthcare access, migrants had lower or similar use of primary health care, mental healthcare or other specialized care than natives; however, a limitation of these studies was that investigators did not take into account the diversity of the migrant community.

Professionals' self-perceived knowledge regarding migrants' health rights was reported as low or limited in more than 60% of respondents, and only 29% reported having acquired such knowledge in the workplace. Our results are similar to those found in a study based in Finland (30), which reported low levels of perceived knowledge of migrants' health rights among professionals. Interestingly, while professionals from the Finnish study ranked the importance of having such knowledge as very high, results from our study suggest otherwise. Only 27% of our participants determined that training professionals to be better versed in health access legalese was a useful strategy. Instead, our results suggest that training efforts ought to be centered around improving intercultural competence of healthcare providers (63%) and/or improving health-system navigation skills for migrants (48%).

Almost all professionals (94%) agreed that migrants face considerable barriers when accessing health services in Spain, even though Spain is considered to uphold a tradition of universal access to healthcare (31). The top five most highlighted barriers perceived by professionals in our study included language (88%), cultural differences (70%), administrative issues (68%), and fear of being undocumented (57%). Despite LA being the largest non-native community in Spain, they only represented 18% of the migrant community in 2018 (32), which may explain why language remains the most important barrier perceived by professionals. Our findings also echo what other researchers have found in the past. For instance, results from two qualitative studies (33, 34) carried out in Catalonia about the needs and perceptions of healthcare providers on the provision of healthcare to migrants, suggested that communication barriers and low levels of cultural competency among professionals were the largest contributors to poor outcomes. Similarly, in a separate study of health-access barriers facing migrants with HIV (35), researchers found communication barriers were the main obstacle facing migrants of non-Spanish speaking countries.

Administrative issues and stigma were also identified as important barriers in our study, although to a lesser degree (30%). These barriers could lead to delays in diagnosing and treating different diseases that carry not only individual, but important public health consequences such as tuberculosis, HIV, hypertension, diabetes, hepatitis B and C, schistosomiasis, strongyloidiasis, and Chagas (36). For example, in the previously mentioned study of migrants with HIV (35), 14–25% of migrants who reported barriers to healthcare access had been diagnosed with late HIV. Furthermore, increasing migrant engagement with preventive health initiatives such as vaccination and/or LTBI screening campaigns may confer benefits on them and the community (5, 37–39). As stated by the WHO, “rapid access to healthcare can result in cure, it can avoid the spread of disease; it is therefore in the interest of both migrants and the receiving country” (40).

We found that professionals perceived mental health disorders as the most common diseases among newly arrived migrants (67%). Our findings resonate with evidence showing that migrants and refugees consistently report higher levels of psychosomatic distress and post-traumatic stress disorder (PTSD) when compared to that of the general population (41). Regrettably, despite the higher reported mental health burden, in a study that assessed the health status of 303 newly arrived asylum seekers in Spain, only 3% of this population were referred to a transcultural psychiatry consultation (36).

Only 15% of the interviewed professionals considered migrants to be generally healthy, even though previous studies have shown that the prevalence of infectious diseases, non-communicable diseases, and specific mental health conditions such as depression and psychosis are generally similar or even lower to that of the general population in the host country (36).

Based on participants opinion, the strategies most highlighted to improve care for migrants and their access to health centers were those related to cultural and language competencies. According to Betancourt et al. (42), “Cultural competence in healthcare entails: understanding the importance of social and cultural influences on patients' health beliefs and behaviors; considering how these factors interact at multiple levels of the healthcare delivery system (e.g., at the level of structural processes of care or clinical decision-making); and, finally, devising interventions that take these issues into account to assure quality healthcare delivery to diverse patient populations" (42). Indeed, providing adequate intercultural competence training for healthcare providers ought to make healthcare services more accessible, acceptable, and effective for people from diverse ethnocultural communities. The progress at which a person or institution acquires cultural competence varies, depending partly on the experiences acquired over time (43). Regretably, the absence of intercultural competence training modules within the official curriculae of social and health sciences of most Spanish university programs may delay or even prevent its development.

While technologically-driven solutions such as mobile applications where ranked as lowest in importance by our participants (7.5%), it is worth mentioning that multiple promising apps that facilitate simultaneous language translation exist, such as Google translator© or Universal doctor©, and are becoming increasingly available to healthcare providers. Perhaps our results reflect the fact that most of these tools are still not mature enough to be implemented at a larger scale within the healthcare or social care settings, as in some cases, migrants may stand as simple listeners. Similarly, the Catalan government provides translators to healthcare professionals with a hotline (061 CatSalut Respon), although the service has important limitations such as the time it takes to find a proper translator once activated, and the restricted time allocated for consultation. In addition, minority languages are sometimes not available, suggesting that the service, while comendable, is still severly limited.

Increasing the role of community health agents was the second most important strategy reported (61%) by our participants. Community health agents are individuals who work to strengthen links between the community and health services. Such agents are not usually certified and carry out their duties outside of national healthcare services. Non-health agents also form part of this collective and focus on social determinants of health, such as housing, inequalities, education, employment or the environment (44). Although the presence of community health agents in health and social centers is a strategy encouraged by the WHO (45), such line of work is not prevalent in Spain and Europe. Some studies, however, suggest that these agents have a positive impact on adherence to and completion of medical follow-ups, as well as on reductions in hospital readmissions in vulnerable populations (44, 46, 47).

### Limitations

While our study represents a unique attempt to define the health needs and barriers facing newly arrived migrants in Spain from the perspective of healthcare and social care providers using a cross-sectional, survey-based methodology, it is not without several limitations worth mentioning. First, our relatively small sample size and the non-probability sampling strategy we used hinder our ability to extrapolate our results to the broader viewpoint held by professionals across Spain. Consequently, the barriers and potential solutions expressed by professionals in our study are likely to represent the unique challenges each participant face in their respective entities, which may or may not correlate to the realities experienced elsewhere. Second, while we tried to follow most standard procedures when it comes to survey development and validation, we did not carry out a proper statistical analysis of our end-survey, so questions regarding the internal structure and reliability of the questionnaire from a psychometric point of view remain unanswered. However, given the exploratory nature of this study, we are confident that our approach to survey development has been adequate, while recognizing that future studies require further testing. Similarly, given our sampling limitations, we decided not to carry out a more in-depth bivariate analysis of our results, which could have painted a more nuanced picture regarding differences among our respondents in terms of professions (e.g., healthcare vs. social care professionals) and gender, for example. In addition, we did not collect professionals' geographical location, and are consequently unable to answer whether such perceptions vary from one geographical area to the next. Third, while we asked participants to focus their attention on newly arrived migrants, we recognize that a migrant population is incredibly heterogeneous, with different geographical and cultural origins, age groups, socioeconomic stratums, and, consequently, may experience important unaccounted health-access and quality of care barriers unique to their group. Lastly, while we tried to address questions regarding face-validity during the questionnaire development process, the online nature of our survey made it impossible for us to ensure that all participants shared a common understanding of some of the surveys' items.

## Conclusion

In conclusion, most healthcare professionals perceive newly arrived non-EU-27 migrants as having worse health status, present higher burden of mental health disorders, and are less prone to abusing healthcare services compared to the native population. Similarly, healthcare professionals perceive themselves as having inadequate knowledge of migrants' health rights, yet most agree that interventions in this regard are better aimed at the migrant community itself. Finally, healthcare professionals agree that language and low levels of cultural competence are among the most important health-access barriers facing newly arrived migrants.

Taken together, our results underline the importance of designing evidence-based interventions that take into account the unique perceptions and experiences of healthcare professionals that engage with newly arrived migrants in their daily practice. While in the last couple of years several such initiatives have been implemented (48, 49) the scarcity of published literature testament of a broader gap. We hope that our study contributes to bridging this gap.

## Data Availability Statement

The raw data supporting the conclusions of this article will be made available by the authors, without undue reservation.

## Ethics Statement

The studies involving human participants were reviewed and approved by Clinical Research Ethics Committee (CEIC) of the Vall d'Hebron Hospital in Barcelona. Written informed consent for participation was not required for this study in accordance with the national legislation and the institutional requirements.

## Author Contributions

NS-D, AD, and IO: conceived of the presented idea. NS-D, AD, BT, and IO: developed the survey and made the recruitment. NS-D, AM-C, EE, and EH: organized the database. NS-D, AM-C, RM, and SA: verified the analytical methods. NS-D, AM-C, and RM: wrote the first draft of the manuscript. All authors contributed to the article and approved the submitted version.

## Author Disclaimer

The content of this article is the sole responsibility of the authors and does not necessarily represent the views of any of the sponsoring organizations, institutes, or the European Commission.

## Conflict of Interest

The authors declare that the research was conducted in the absence of any commercial or financial relationships that could be construed as a potential conflict of interest.

## Publisher's Note

All claims expressed in this article are solely those of the authors and do not necessarily represent those of their affiliated organizations, or those of the publisher, the editors and the reviewers. Any product that may be evaluated in this article, or claim that may be made by its manufacturer, is not guaranteed or endorsed by the publisher.
