# Supplementary material for: A Cross-Sectional Survey on Professionals to Assess Health Needs of Newly Arrived Migrants in Spain
Source: Front Public Health. 2021 Aug 2;9:667251. doi: 10.3389/fpubh.2021.667251 (PMC8365167; doi:10.3389/fpubh.2021.667251)
Supplement: Supplementary file 1 [file Data_Sheet_1.docx]

Annex 1 INTERVIEW FOR HEALTHCARE PROFESSIONALS

1. Interview Date…..…./…..…../2018
2. Professional: Administrative Nursing Medical Non-health related
3. Years working: <5 years 5-10 years >10 years
4. Sex: Female Male
5. Birth country: __________________
6. Country where you work: ______________
7. Birth year: ____________________
8. Type of center (indicate): Hospital / Primary Health Care / ΝGO/ Foundation or Association/Other (please specify)
9. What percentage of the population who attends your center are migrants? (indicate):

<5 % 5-10% 11-20% 20-40% 40-99% 100%

1. Which community/communities do you think is/are most represented among the users of your center? (You can select more than one option)
   1. sub-Saharan African
   2. subcontinent Indian (India, Pakistan)
   3. Latin American
   4. North African
   5. Eastern European
   6. Middle Easterner
   7. Other……………………………………………………………
2. From 1 to 10, what do you think is the average health level of the population where you live?

Very low 1—2—3—4—5—6—7—8—9—10 Excellent

1. From 1 to 10, what do you think is the average health level of the migrant population?

- sub-Saharan African: very low 1—2—3—4—5—6—7—8—9—10 Excellent
- subcontinent India (India, Pakistan): very low 1—2—3—4—5—6—7—8—9—10 Excellent
- Latin American: very low 1—2—3—4—5—6—7—8—9—10 Excellent
- North African: very low 1—2—3—4—5—6—7—8—9—10 Excellent
- Eastern European very low 1—2—3—4—5—6—7—8—9—10 Excellent
- Middle Easterner: very low 1—2—3—4—5—6—7—8—9—10 Excellent
- Other: very low 1—2—3—4—5—6—7—8—9—10 Excellent

1. What do you think is the level of use of health services by:

- Native Spanish: Little use Adequate use Abusive use
- Of immigrants, refugees, and asylum seekers: Deficient use Adequate use Abusive use

1. What level of knowledge do you have about the degree of healthcare coverage (rights) for refugees/immigrants and asylum seekers in your country/region?

Adequate knowledge Limited knowledge Little knowledge

1. How have you acquired knowledge about immigrant coverage? (You can select more than one option)

- I have received specific training in my workplace
- Through co-workers informally
- Through social media
- Self-taught
- Other form; specify………………………………………………………………………………………
- I do not have any knowledge

1. Do you think there are barriers/problems/difficulties for the migrant population when accessing health services?

Yes No I don’t know

1. If you answered “Yes” to question 16, what barrier/problems/difficulty was/wrere it? (You can mark more than one answer)

Cultural Language

Religious Administrative

Work or employment Geographic

Fear of being undocumented For being a man or woman

Family obligations Stigma/prejudices

Other (Please specify)

1. In your opinion, what type of disease is more frequent among immigrants, refugees, and asylum seekers? (you can select 3 options maximum)

Chronic disease (obesity, hypertension, diabetes…)

Anxiety/stress/difficulty sleeping/difficulty concentrating

Infectious diseases (HIV/AIDS, sexually transmitted infections, viral hepatitis, tuberculosis.)

Dental health

Other

In general, they are healthy

Do not know

1. From 1 to 10, what do you think is the quality of care (social and health) provided to migrants, refugees, and asylum seekers in your area?

Very low 1—2—3—4—5—6—7—8—9—10 Excellent

1. Have you received specific training on social or healthcare for the immigrant population?

Yes No Don’t know

1. What tools/strategies do you think could improve care for immigrants, refugees, and asylum seekers in your center? (You can select 3 options maximum)

- Translators
- Community health agents
- Training of socio-health personnel on intercultural health skills for the immigrant population
- Training of socio-health personnel on diseases prevalent in country of origin
- Specific training for immigrants, refuges, and asylum seekers on using the health system
- **Workshops/education activities and health promotion for immigrant population in vulnerable situations**
- Training on the decree that regulates access to the health system in these groups
- Specific referral circuits for tropical medicine or international health services
- Specific referral circuits for mental health
- Mobile applications/internet with updated information
- Other………………………………………………………………………………………………

1. Finally, do you think this questionnaire that you have answered helps to better understand problems faced by some immigrant groups when accessing health services (primary healthcare and hospitals)? Please mark from 1 to 10.

A little 1—2—3—4—5—6—7—8—9—10 Extremly
